# Association between human cercarial dermatitis (HCD) and the occurrence of *Trichibilarizia* in duck and snail in main wetlands from Mazandaran Province, northern Iran

**DOI:** 10.1016/j.parepi.2021.e00211

**Published:** 2021-03-16

**Authors:** Elham Kia lashaki, Shirzad Gholami, Mahdi Fakhar, Mehdi Karamian, Ahmad Daryani

**Affiliations:** a Molecular and Cell Biology Research Center, Department of Parasitology, Faculty of Medicine, Mazandaran University of Medical Sciences, Sari, Iran; b Toxoplasmosis Research Center, Communicable Diseases Institute, Iranian National Registry Center for Lophomoniasis and Toxoplasmosis, Mazandaran University of Medical Sciences, Sari, Iran; c Zanjan University of Medical Sciences, Department of Medical Parasitology and Mycology, Zanjan, Iran; d Toxoplasmosis Research Center, Communicable Diseases Institute, Department of Parasitology, Faculty of Medicine, Mazandaran University of Medical Sciences, Sari, Iran

**Keywords:** Bird schistosomes, Domestic duck, Freshwater snail, Cercarial dermatitis, Wetlands

## Abstract

**Background:**

Avian schistosomes are considered as main causative agents of human cercarial dermatitis (HCD) in Iran. The study was conducted to determine bird schistosomes in their final and intermediate hosts, in main wetlands of Mazandaran.

**Methods:**

A total of 255 domestic and wild ducks were collected and the infection of nasal tissues of five (*Anas platyrhynchos domesticus*, *Aythya ferina, Cairina moschata, Anas platyrhynchos* and *Spatula clypeata*) species were analyzed using morphological techniques. Also, 1687 freshwater snails were collected and surveyed by cercarial shedding and crushing tests. Detection of HCD was performed for the presence of clinical symptoms of itching and maculopapular rashs by physical examination.

**Results:**

Of 255 ducks, in 41 (16%) infection with nasal *Trichibilarizia* spp. were recognized by observing eggs and/or adult worms. The most infected ducks were *Anas clypeata* and *Anas platyrhynchos domesticus*. Overall, 0.17% of snails were infected with avian schistosomes. Also, clinical examination of 951 rice farmers revealed that 588 (61.82%) of them were suffered from HCD.

**Conclusion:**

Our data suggest that domestic ducks could play a prominent role as a reservoir host for establishing life cycle of *Trichobilharzia* in the area. Also, existence of domestic reservoir ducks and suitable snail hosts in ponds and paddy fields of this area, climate conditions of the wetlands in Mazandaran leads to a high incidence of HCD.

## 1 Introduction

Study on avian schistosomes is very important not only from a veterinary perspective but also as a means of predicting the hazard of human infection by the schistosome cercariae (Picot et al., 1996; Lashaki et al., 2020; Żbikowska, 2002). This infection known as human cercarial dermatitis (HCD), regarded as a re-emerging infectious disease in human (Kolářová et al., 2013). The disease is an inflammatory reaction happening in the skin of persons who have contact with the water. It seems that HCD is a worldwide, neglected parasitic skin disease (Kolářová et al., 2013). The relation between bird schistosomes and HCD has been recognized for a long time (Horák et al., 2015). Overall, global prevalence of avian schistosomes was estimated to be 34.0%. In addition, results of a systematic review showed that, *Allobilharzia visceralis* and *Trichobilharzia* spp. had the highest global prevalence in birds (Lashaki et al., 2020). However, bird schistosomes are considered as the most neglected of the neglected zoonotic parasitic worms among waterfowls (Lashaki et al., 2020).

Bird schistosomes have a two-host life cycle including water snails and birds as intermediate and final hosts, respectively. Among bird schistosomes, members of the genus *Trichobilharzia* are *categorized into* visceral and nasal groups depending on their target tissue within the final hosts (Horák et al., 2015). *Trichobilharzia regenti* is a neurotropic avian schistosome that can use a diversity of definitive bird hosts and *Radix* snails as the intermediate hosts throughout Eurasia region (Leontovyč et al., 2016). HCD seems to be distributed along the main flyways of migratory birds that transport the schistosomes to and from wintering regions and then spreading this agent in the water resources which suitable intermediate hosts' habitats located in them (Żbikowska, 2002; Horák et al., 2015). In the last decade, we have seen an increase in reported cases of HCD, especially in Iran (Farahnak and Essalat, 2003; Rostami-Jalilian, 2006; Karamian et al., 2011; Gohardehi et al., 2013; Maleki et al., 2012; Mahdavi et al., 2013; Rahimi Esboei et al., 2013; Ashrafi et al., 2018). The persistent contact of rice farmers in paddy field, in northern Iran, with water containing cercariae has made HCD an endemic disease in this region (Rostami-Jalilian, 2006; Rahimi Esboei et al., 2013).

Various parts of Mazandaran Province, northern of Iran are proper places for wintering of the migratory birds. Every year thousands of birds migrate to the natural habitats of this province. These migratory birds shed eggs that hatch and expose the snails to miracidia, and contaminate snails. These snails release cercaria in water and cause illness in individuals who are in contact with fresh infected waters. HCD is a zoonotic disease that has thus far been reported from the northern (Mazandaran and Guilan) and southern (Khuzestan) provinces in Iran (Farahnak and Essalat, 2003; Ashrafi et al., 2018). Studies on migratory ducks from Mazandaran province have reported avian schistosomes (Rostami-Jalilian, 2006; Mahdavi et al., 2013; Rahimi Esboei et al., 2013) and Fakhar et al., 2016 reported that one of the most prevalent species found was a neuropathic species, *Trichobilharzia* cf. *regenti* (Fakhar et al., 2016). Studies in a neighboring province reported that domestic ducks acted as reservoir hosts in that area (Ashrafi et al., 2018), but no effort has been made in the wetland areas of Mazandaran province to estimate prevalence in domestic ducks.Thus, the question here is whether or not domestic ducks in the region are reservoir hosts for the neuropathic species and if so, what is the prevalence of HCD among rice farmers? In addition to the examination of domestic ducks and survey of rice farmers for HCD, potential gastropod hosts were collected to report the presence or absence of bird schistosome cercariae.

## 2 Materials and methods

### 2.1 Ethics approval and consent to participate

The current study was approved by the Ethical Committee of the Faculty of Medicine, Mazandaran University of Medical Sciences, Sari, Iran (IR.MAZ.REC.1397.1692).

### 2.2 Examination of migratory ducks

The study was conducted in Mazandaran Province in northern Iran. This province is bordered clockwise by Russia (across the sea) and the provinces of Golestan, Semnan, Tehran, Alborz, Qazvin and Guilan. It has a humid and subtropical climate with an average temperature of 17 °C. This province is an ecotourism area which attracts many travelers every year (Mesgari et al., 2013)*.* Three important wetlands (Seyed Mahale, Sorkhrud and Ezbaran, 536¢E, 3623¢N) were selected to investigate the prevalence of nasal avian schistosomes. In the plains of these areas, the main occupation is rice cultivation and water for agriculture is supplied by man-made ponds that have water year-round. Therefore, these places are suitable for migratory and resident birds. In the present study, a total of 95 heads of *migratory aquatic birds* (Anatidae; *Anas platyrhynchos*, *Spatula clypeata*) were legally bought from the local hunters of Mazandaran districts, between January and March 2018. The samples were transported to the Helminthology Research Laboratory at Mazandaran University of Medical Sciences for parasitological examination and were kept frozen until examination. The heads of birds were examined for nasal schistosomes by dissection of the nasal tissue according to procedure by Skírnisson and Kolářová (Skírnisson and Kolářová, 2008). The presence of adult schistosomes and their eggs were surveyed by microscope at low (10×) magnification.

### 2.3 Examination of domesticated ducks

Between May and June 2018, a total of 160 domesticated birds from the study area were examined for the presence of nasal bird schistosomes. The ducks were collected from local markets at different localities in the Province (Sari, Sorkhrud, *Mahmudabad and* Fereydoon Kenar) and belonged to 3 species: *Anas platyrhynchos domesticus*, *Aythya ferina* and *Cairina moschata.* The stages of dissection and examination were mentioned above.

### 2.4 Examination of snails

To detect ocellate schistosome cercariae, a total of 1687 freshwater snails were collected by hand from three wetlands (Seyed Mahale, Sorkhrud and Fereydoon Kenar) of the province, between March and June 2018. The snails were collected by hand and transported to the parasitology research laboratory of Mazandaran University of Medical Sciences. At first, all snails were counted. Species of the snails were identified with the help of diagnostic keys provided by Mansoorian (Mansoorian, 1986; Pfleger, 1999). According to species, every 10 snails were put in separated tubes comprising dechlorinated tap water and placed under artificial light for at least 2 h or overnight to induce cercarial shedding (Faltýnková et al., 2008). Snails from positive tubes were tested individually under the conditions mentioned above. If no cercarial shedding were observed, the snails were compressed and crushed between two glass slides (25 × 75 mm) and studied by stereo microscope. The collected furcocercous cercariae with pigmented eye spots were identified by systematic key references (Frandsen and Christensen, 1984; Cichy and Żbikowska, 2016).

### 2.5 Examination of cercarial dermatitis

Between March and June 2018, a total of 951 rice farmers were examined by physical examination for the presence of clinical symptoms of itching and maculopapular rashs or skin erosions as well as clinical history. The clinical signs and symptoms of the patients were recorded.

## 3 Results

### 3.1 Nasal schistosomes in migratory ducks

**Fig. 1:**
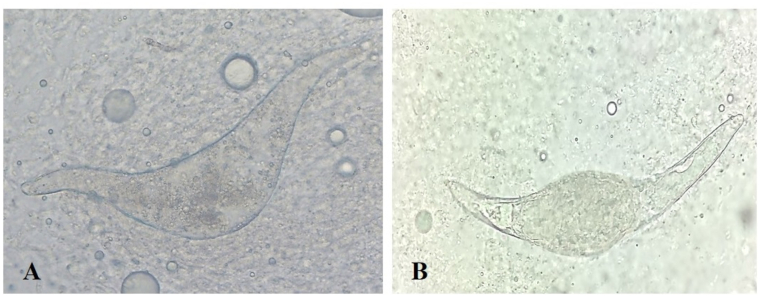
Egg of Trichobilharzia. (A) An undeveloped/dead egg of *Trichobilharzia* from nasal tissues of *Anas platyrhynchos*; (B) an egg with a miracidium.of *Trichobilharzia* from nasal tissues of *Anas platyrhynchos domesticus* (original magnification ×40).

The examination of the migratory ducks (Table 1) showed that 17 out of 95 (17.8%) birds were infected by *Trichobilharzia* sp. Eggs and/or adult fragments were found in 10/16 (62.5%) *S. clypeata* and 7/79 (8.8%) *A. platyrhynchos* (Table 1). The prevalence of *Trichobilharzia* sp. in *S. clypeata* was significantly higher than *A. platyrhynchos* (*P* < 0.05).The infection of the birds in this study was recognized by observing eggs and/or adults of avian schistosomes in their nasal tissues. Certain spindle shape of the eggs indicates the infection of birds with *Trichobilharzia* sp. (Fig. 1A). Fragments of adults were seen in the nasal mucosa of two *Anas platyrhynchos*.

**Table 1:** Prevalence of nasal *Trichobilharzia* in migratory and domesticated ducks in the studied area.

Bird species	No. of examined	No. of infected	Prevalence (%)
Domesticated ducks			
*Anas platyrhynchos domesticus*)	103	19	18.44
*Aythya ferina*	42	5	11.9
Muscovy duck(*Cairina moschata*)	15	–	–
Total	160	24	15

Migratory ducks			
*Anas platyrhynchos*	79	7	8.8
*Anas clypeata*	16	10	62.5
Total	95	17	17.8

### 3.2 Nasal schistosomes in domesticated ducks

Of the 160 studied domesticated ducks, 24 (15%) were found infected with eggs of nasal *Trichobilharzia* (Table 1). All the infected birds belonged to *Anas platyrhynchos domesticus* and *Aythya ferina*. Eggs of *Trichobilharzia* sp. were found in 19 out of 103 (18.44%) *Anas platyrhynchos domesticus*, while 5 out of 42 (11.9%) *Aythya ferina* examined were infected by theses schistosomes. The eggs of these species of schistosomes are spindle-shape (Fig. 1 B).

### 3.3 Infected freshwater snails

A total of 1687 freshwater snails collected from several locations of the Province were examined. Abundance of these snails was *Physa acuta* 54.5%, *Lymnaea stagnalis* 23.1%, *Lymnaea gedrosiana* (= *Radix auricularia*) 18.1% and *Planorbis planorbis* 4.1% (Table 2). Another point to note is that the sequencing of ribosomal DNA ITS-2 indicated that *Lymnaea gedrosiana* (= *Radix auricularia*), the haplotype introduced in Bandar-Anzali, north of Iran, being similar to that recorded in several European countries (Dodangeh et al., 2019).

**Table 2:** The prevalence of snails infected with *Trichobilharzia* spp. cercariae in wintering regions for migratory birds, in the north of Iran.

Snail species	No. examined	No. infected	Percentage (%)
*Radix auricularia*	306	2	0.65
*Lymnaea stagnalis*	391	1	0.25
*Physa acuta*	920	–	–
*Planorbis planorbis*	70	–	–
Total	1687	3	0.17

The parasitological examination of cercariae isolated from these snails revealed that 3 snails (0.17%) were infected with ocellate furkocercariae. The most infected snail was *R. auricularia* (0.65%). Also, infection rate in L. *stagnalis* was 0.25%.

### 3.4 HCD cases

**Fig. 2:**
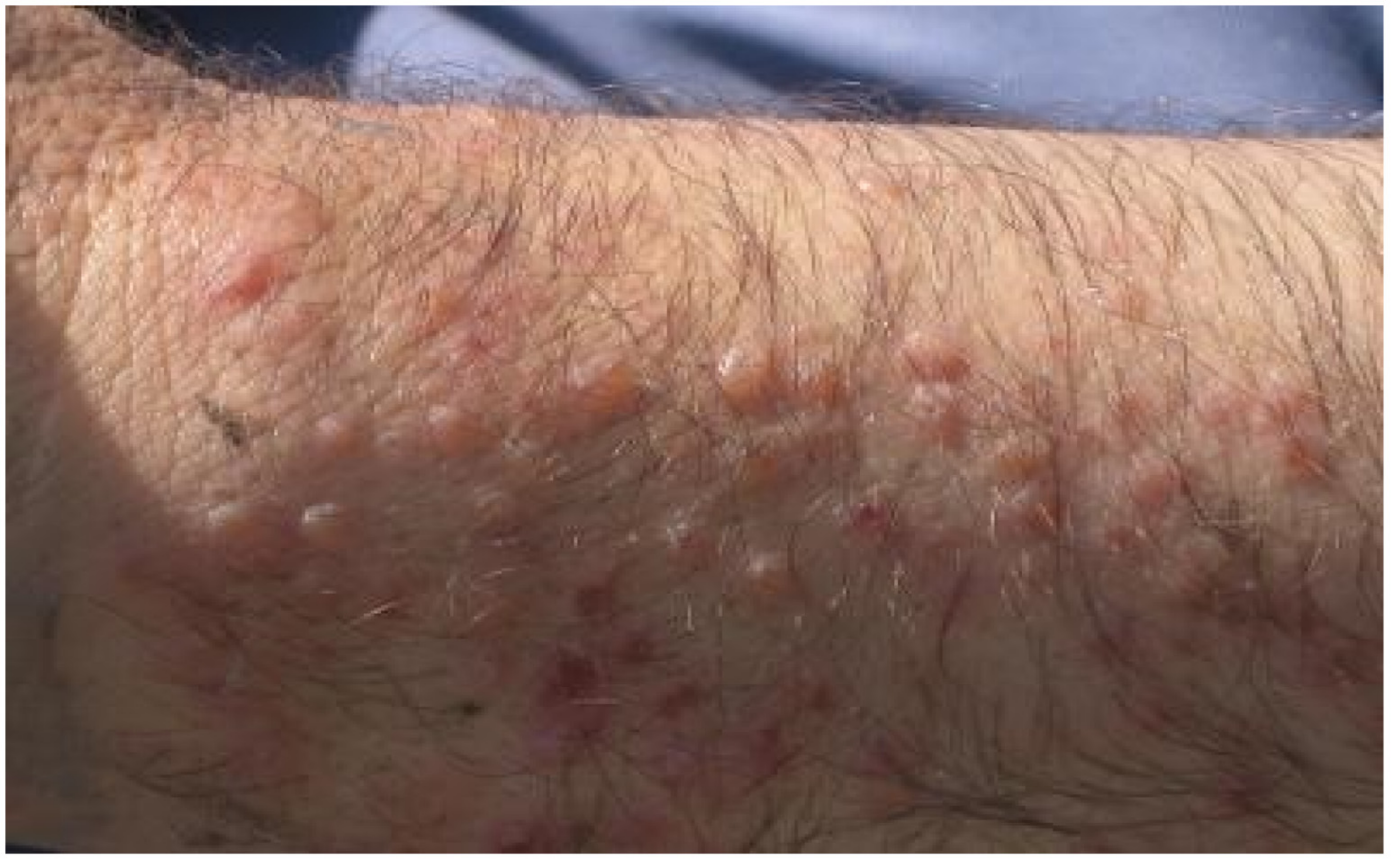
Rashes on the hands of a farmer in Fereydoon Kenar town (north of Iran).

Of the 951 examined rice cultivation workers, 588 (61.82%) had macular or popular rashes on their hands (37%) and feet (63%) (*P* > 0.05) (Fig. 2). Based on the history of the disease, symptoms started with erythematic features, itching, and several hours later, maculopapular rashes became visible. The most of farmers were adult and they were living in the local districts. In the present study, variables such as gender, past history of HCD, affected body part and intensity of infection were also evaluated. The data show that 68% of the infected farmers had a past history of HCD, the severity of infection in majority of patients (62.24%) was severe and 42.5% of the infected subjects were female. Due to the lack of cooperation of the farmers studied, more information is not available.

## 4 Discussion

The results of the present study indicated that nasal avian schistosomes are frequent parasites of Anatid birds in Mazandaran Province, Northern Iran. In this study, the infection rate of migratory and domestic ducks by nasal avian schistosomes were 17.8% and 15% respectively. The prevalence of nasal avian schistosomes in migratory ducks was higher than native ducks. In our study, the prevalence of nasal *Trichobilharzia* in migratory birds was 62.5% for *S. clypeata* and 8.8% for *A. platyrhynchos*. *S. clypeata* had the highest infection rate with avian schistosomes among migratory ducks. In the study of Fakhar et al. (2016) the prevalence of nasal *Trichobilharzia* was 32.6% for *S. clypeata* and 8.6% for *A. platyrhynchos*. Thus, the prevalence of nasal schistosomes in *S. clypeata* was remarkably higher than in *A. platyrhynchos* (Fakhar et al., 2016). In two previous studies conducted in the Mazandaran Province area, Gohardehi et al. (Gohardehi et al., 2013) reported a higher prevalence of infection by nasal *Trichobilharzia* in *S. clypeata* than in *A. platyrhynchos* and Maleki et al. (Maleki et al., 2012) reported the prevalence of nasal *Trichobilharzia* in *S. clypeata* as 33.3%. These investigations, like our research, were conducted during the hunting season (winter). The prevalence values could show that *S. clypeata* is the main host, or at least the host that spends the most time in contact with the snail host.

In European countries such as Germany, the prevalence of nasal *Trichobilharzia* was reported 21% examining migratory *Anas plathyrhynchos* (Prüter et al., 2017).Also, in the Czech Republic, Rudolfova et al. emphasize the importance of *A. platyrhynchos* as definitive host for nasal schistosomes of wildfowl in this area (Rudolfova et al., 2007).

These studies show that *A. platyrhynchos* and *S. clypeata* are common hosts for nasal *Trichobilharzia*().These birds are a surface-feeding duck who feeds in shallow parts of the water. Due to their phototactic behavior, the cercariae reach the surface water and thus, the probability of exposure to cercariae increases for these species of birds (Blair and Islam, 1983).

Previous studies on avain schistosomes in Mazandaran Province focused only on migratory birds (Fakhar et al., 2016); but in the present study for the first time we report nasal schistosomes of both domestic and migratory ducks in this region. In fact, our present work, along with previous studies conducted in the area (Ashrafi et al., 2018; Fakhar et al., 2016), was able to complete main part of the *Trichobilharzia* life cycle puzzle (snail and ducks) in the region. Various regions of the Mazandaran Province are suitable places for the winter migratory birds. Every year thousands of birds migrate to the northern provinces of Iran from the Central Europe and Russia (Jouet et al., 2009; Behrouzirad, 1994). This region is a major stopover for migratory birds and also has a high incidence of HCD, particularly in rice farms (Rahimi Esboei et al., 2013; Pfleger, 1999). According to the results of this study, infection with avian schistosomes is endemic in wild and domestic ducks of Mazandaran Province, so migration of waterfowl toward the northern parts of Iran can transmit and distribute avian schistosomes in their migratory routes in the environment. Close association of wild ducks, domestic ducks and intermediate snails of avian schistosomes in water resources, provide a persistent risk of exposure to HCD for farmers in this region (Ashrafi et al., 2018; Fakhar et al., 2016).

In the present study, high prevalence (15%) of *Trichobilharzia* in domestic ducks was observed in Mazandaran Province. All the infected birds belonged to *Anas platyrhynchos domesticus* and *Aythya ferina*. The highest infection prevalence was found in *Anas platyrhynchos domesticus* (18.44%). Researchers reported a 32% prevalence of *Trichobilharzia regenti* in the nasal cavities of *Anas platyrhynchos domesticus* in Guilan province (Ashrafi et al., 2018).

In our research, the prevalence of infection in *Aythya ferina*, one of the most frequent domestic ducks in this region, was 11.9%. However, in present study, no schistosomes were obtained from *Cairina moschata*. These ducks rarely swim, because their sebaceous glands are less developed than other ducks (Rudolfova et al., 2007) and are likely to be less infected with *Trichobilharzia* cercariae as a result of less water contact.

Among migratory ducks, *Aythya ferina* and *Anas platyrhynchos* have been domesticated over time and bred in different parts of Mazandaran Province. Many hunters and farmers are residents around ponds and rice fields. They bred domesticated ducks in these areas for the purpose of selling on the market or as bait for hunting migratory birds. Our results support that domestic ducks same as migratory ducks play a significant role in establishing life cycle of *Trichobilharzia* in the region. As Ashrafi et al. in Guilan province showed that the domestic duck) *Anas platyrhynchos domesticus(*can serve as a reservoir host for *T. regenti* and that one of the native snail species is probably the intermediate host (Ashrafi et al., 2018).

In the present study, a small percentage of freshwater snails (0.17%) infected with *Trichobilharzia* cercariae. The overall infection rate is less than 1.2% prevalence rate which was reported by Gohardehi et al. (2013) who examined 676 snails from paddy fields in Mazandaran province (Gohardehi et al., 2013). Similar study conducted in this region by Rostami-Jalilian (2006) indicated a lower infection percentage (0.05%) among the surveyed snails compared to our study. In this investigation, most schistosomes' infections were recorded from *R. auricularia* (0.65%) and no schistosome found from *Physa* spp. Our results support Gohardehi et al. (2013) findings that showed that the most infected snail species was *R. auricularia* and none of physid snails were infected with furcocercariae of avian schistosomes. So, it seems that lymnaeid snails are the suitable intermediate hosts in Iran. Different species of Lymnaeidae such as *Stagnicola palustris*, and *Radix auricularia* have been reported from Guilan Province and are potential hosts (Ashrafi and Mas-Coma, 2014; Ashrafi et al., 2015).The small number of Planorbis collected in this study, compared with the results of Rostami-Jalilian et al. investigation can justify the absence of infection of these snails with avian schistosomes (Rostami-Jalilian, 2006). Also, based on the systematic review and meta-analysis performed by Dodangeh et al.‚ the highest rate of infection with larval stages of the trematodes was observed in *Radix auricularia* (9.9%) (Dodangeh et al., 2019).

This difference in the prevalence of infection in snails may be influenced by sampling season, age and size of collected snails. An investigation by Graham (Graham, 2003) showed that the size and age of a snail are positively correlated with the rate of infection; snails of different sizes may have experienced different duration of exposure to the schistosomes miracidia and development of infection. Unfortunately, in our research, this information is not available.

In spite of the relatively low prevalence of avian schistosome cercariae in the studied region, there were many cases of HCD in the inhabitants of these areas. Factors like the release of a large number of cercariae by infected snails and lasting viability of cercariae can justify the high level of infection in the area (Żbikowska, 2004).

According to this study, there are two sylvatic and domestic cycles for the causative agent of HCD in this region. Although the infection rate is higher in the sylvatic cycle involving wild migratory ducks, but the migrant birds are temporarily staying in Iran. In the domestic cycle, there are two species of ducks that can be infected by nasal avian schistosomes. Considering the climate, geographic conditions and the jobs of the region's inhabitants, the presence of suitable reservoir bird hosts and the existence of lymnaeid snails in these rice fields have the potential to infect the people of this region.

In this study, a population of 951 rice farmers was studied in the Mazandaran Province and it was found that 68.6% of them had HCD. In a previous survey by Rahimi- Esboei et al. 77.5% of the rice workers studied in Mazandaran Province suffered from HCD (Rahimi Esboei et al., 2013). But in the study conducted by Farahnak et al. in Khuzestan Province, 1.1% of investigated inhabitants were infected by the cercarial dermatitis agents (Farahnak and Essalat, 2003). Due to high humidity, more rainfall and winter migration of birds to Mazandaran Province, high prevalence of infection in domestic ducks and their breeding in paddy field, HCD is more prevalent in this area. On the other hand, because livestock breeding in the studied areas is not common, however mammalian schistosomes such as *Schistosoma turkestanicum* have been recently recorded (Sahba and Malek, 1979). Hence bird schistosomes are considered as main causative agents of HCD in the studied area.

As noted in the results, the highest rate of infection was observed among female farmers.

In our study the incidence of HCD in female farmers was higher than male as a result of several possible factors such as differences between skin structure and surface lipids of women than men, role of sex hormones like estrogen (Vom Steeg and Klein, 2017), also women are frequently employed as cheaper workers in paddy fields in the region, hence they are more contacted with water. Thus women are likely to be more susceptible to HCD and have a higher chance of getting infected in the area.

Since we do not know about *Trichobilharzia* species in ducks and snails in our research, molecular studies are needed in migratory, native and snail ducks. Our study shows in which areas the precise determination of species should be done in order to carry out control measures and epidemiological studies. Also, in this study, only nasal schistosomes were studied, but efforts need to be made to identify the other species of schistosomes in wild and domestic ducks.

In conclusion, infections by the nasal schistosomes occur frequently in the Mazandaran Province in both domesticated and migratory ducks. This survey presents the first report of nasal *Trichobilharzia* in domestic ducks, in the Mazandaran Province which identified by morphological methods. Our result suggest that domestic ducks could play a prominent role as a reservoir hosts for establishing life cycle of *Trichobilharzia* parasite in the area. Also, the present study indicates that HCD is a common health problem in the research zone. Consequently, the control measures, such as public education and the use of biological methods should be addressed in the region. A study by Marszewska et al. in European recreational water bodies showed that a non–host snail population lowers the transmission of bird schistosomes miracidia to suitable snail hosts such as *R.balthica*. *Potamopyrgus antipodaruman* (an alien in Europe) could be a good candidate against swimmer's itch because of its apparent resistance to invasion by European bird schistosome species and its high population density (Marszewska et al., 2018). Considering that the most infected organ is the farmer's feet, it is suggested that the wearing of appropriate footwear will prevent the disease. As humans are frequently exposed to these parasites, studies on immunopathological and clinical aspects of infection of these agents are recommended.

## Declaration of Competing Interest

Authors declare that have no competing interests.
